# Correlation of Serum Soluble Interleukin-7 Receptor and Anti-C1q Antibody in Patients with Systemic Lupus Erythematosus

**DOI:** 10.1155/2016/8252605

**Published:** 2016-03-16

**Authors:** Shuhong Chi, Jing Xue, Feng Li, Caixia Zhu, Yunxia Yu, Haibo Li, Xuemei Wang, Yurong Zhang, Jijuan Yang, Shaolan Zhou, Lijuan Yang, Chen Ji, Xiaoming Liu

**Affiliations:** ^1^Department of Rheumatology, General Hospital of Ningxia Medical University, Yinchuan, Ningxia 750004, China; ^2^College of Life Science, Ningxia University, Yinchuan, Ningxia 750021, China; ^3^The Center of Laboratory Medicine, General Hospital of Ningxia Medical University, Yinchuan 750004, China; ^4^Institute of Human Stem Cell Research at General Hospital of Ningxia Medical University, Yinchuan, Ningxia 750004, China

## Abstract

*Background.* Serum concentrations of soluble interleukin-7 receptor (sIL-7R) and anti-C1q antibody have recently been identified as unique serological markers for lupus nephritis (LN) in patients with systemic lupus erythematosus (SLE). In this study, we evaluated the correlation of serum sIL-7R and anti-C1q in SLE patients.* Methods.* Sera from 134 patients with SLE and 84 healthy cohorts were tested for levels of sIL-7R and anti-C1q antibodies in terms of ELISA. Correlations of the sIL-7R and anti-C1q autoantibodies were evaluated.* Results.* The serum concentrations of sIL-7R and anti-C1q antibodies were significantly higher in SLE patients and LN patients in comparison with healthy individuals/controls and SLE patients with non-LN, respectively. In addition, both sIL-7R and anti-C1q concentrations were found to significantly correlate with the SLE disease activity as evaluated by SLEDAI scores. Interestingly, the serum sIL-7R concentration was strongly correlated with the level of anti-C1q antibodies (*r* = 0.2871, *p* = 0.0008) but not statistically correlated with other serological markers, including the anti-dsDNA and complements C3 and C4 concentrations in SLE patients.* Conclusion.* Both serum sIL-7R and anti-C1q antibodies were strongly associated with disease activity and LN in SLE patients, suggesting that they may be reliable serological markers for identification of SLE patients with active diseases and LN.

## 1. Introduction

Systemic lupus erythematosus (SLE) is a chronic autoimmune disease that is able to affect multiple systems and major organs, among which lupus nephritis (LN) is one of the most common major organ manifestations and a main cause of the morbidity and mortality of the disease [1]. An involvement of renal disease activity is thus one of the most important prognostic factors for SLE patients, and an identification of LN in SLE patients has an important clinical implication in guiding treatments for SLE in a clinical setting [2]. Owing to the serological hallmark of aberrant production of a broad heterogenous group of autoantibodies in SLE patients, an evaluation of clinical relevance of these profiles of autoantibodies and disease parameters thus has aided in identifying SLE patients at risk for specific complications at an early stage and enabling clinicians to initiate an effective therapeutic strategy and possibly decrease the morbidity and mortality for SLE patients [1–4].

There are more than 180 autoantibodies that have been reported in SLE patients, among which antibodies (autoantibodies) against complement C1q (anti-C1q) and nuclear (antinuclear antibodies, ANA) and double-strand DNA (anti-dsDNA) spurred the most interests in clinical settings [5]. In this respect, anti-dsDNA and anti-C1q antibodies exhibited a stronger association with clinical features of active SLE, particularly with the renal disease activity, than other serological antibodies, indicating an important value of measuring these autoantibodies in SLE patients [4, 6]. Indeed, SLE patients with both anti-dsDNA and anti-C1q antibodies often had a manifestation of renal disease and poor renal outcome, and an increased serum concentration of anti-C1q antibodies is often accompanied with a decreased serum level of complement C1q in patients with active LN [7, 8]. Serum anti-C1q antibodies are thus considered as a biomarker for prediction of renal flares in SLE and have been extensively studied [6, 7, 9–15]. Of note, in addition to the increased concentration of anti-C1q antibodies, serum levels of complements C1q, C3, and C4 are often decreased in SLE patients [16]. Therefore, combinations of serum levels of C1q, C3, and C4, and/or the autoantibodies to C1q, dsDNA, and chromatin/nucleosome, have been evaluated as important immunological markers for diagnosis of SLE, particularly for LN disease [6, 8, 10–12, 16–18].

In general, SLE is recognized as a disease that is primarily attributed to autoantibodies and immune complex deposition. However, mounting evidence has recently suggested that cytokines are also involved in the pathogenesis of SLE [1, 19]. Cytokines are important soluble mediators of intercellular communication and orchestrate the interaction of immune cells during immune responses, which play crucial roles in the differentiation, maturation, and activation of various immune cells. With respect to SLE, cytokines are key players of general immune dysregulation not only in SLE pathogenesis, but also in the local inflammatory responses that ultimately lead to tissue injury and organ damage [1, 19]. Therefore, cytokines may serve as predictive biomarkers for SLE diagnosis and prognosis, as well as therapeutic targets for disease treatments [20, 21]. Several cytokines have been investigated as biomarkers of SLE manifestations including the LN, among which the interleukin-7 (IL-7)/IL-7 receptor (IL-7R) signaling recently received an increased attention, owing to its strong association with the activity of LN of SLE patients [22–26].

IL-7 has been demonstrated to play a fundamental role in T-cell development, homeostasis, and immune tolerance [27]. Under physiological conditions, IL-7 is controlled in a limited resource, since tonic IL-7 signals can be continuously delivered to T-cells, and provides continuous survival signals to naïve T-cells. This differs from activation cytokines, of which the cytokine production and receptor expression only mediate transient effects following immune activation [28, 29]. Therefore, a reduced IL-7 consumption in lymphopenic hosts sequentially leads to an elevated IL-7 level, which in turn enhances proliferative responses to weak self-antigens and results in a homeostatic proliferation [30]. Several lines of study have recently demonstrated that an increased level of soluble IL-7R (sIL-7R) had clinical implications in autoimmune diseases, including rheumatoid arthritis (RA), multiple sclerosis (MS), and SLE [26, 31–33]. In this context, the circulating sIL-7R binds to IL-7 and competes with the cell-associated IL-7R complex to reduce excessive IL-7 signaling, consequently leads to a deceased consumption of IL-7, and enhances an overall IL-7 bioavailability, since IL-7 is a limited resource whose level is regulated primarily* via* receptor-mediated clearance. In addition, sIL-7R is also able to modulate the quality of the IL-7 signal to decrease the induction of negative regulator [24].

With respect to SLE, involvements of IL-7 and sIL-7R in its disease progression were evidenced by studies of genetic association and assessment of plasma sIL-7R concentration [23, 24, 26, 34]. Polymorphic analysis identified several IL-7R single nucleotide polymorphisms (SNPs) that were associated with the susceptibility to SLE and/or LN in SLE patients [24, 34]. For example, Wang et al. recently examined an association of IL-7R SNP rs6897932 (C/T) with the susceptibility to SLE and found that the major allele C of this SNP was associated with increased SLE risk in Chinese populations, although no significant association of the SNP and the presence of 11 subphenotypes, including the LN, was established [34]. In another study, Lundstrom et al. measured the plasma sIL-7R*α* concentrations between multiple sclerosis (MS) patients with IL-7R^*∗*^CC (autoimmune-predisposing) and IL-7R^*∗*^TT (autoimmune protective) genotypes, and they found about 3-fold higher sIL-7R*α* in MS patients harboring IL-7R^*∗*^CC gene relative to those who had an IL-7R^*∗*^TT genotype [24]. Indeed, several lines of study have recently suggested that an elevated level of plasma sIL-7R in SLE patients was correlated with or predicted the occurrence of an SLE nephritis flare, indicating that the serum sIL-7R concentration may be a potential biomarker with high sensitivity and specificity for diagnosis of SLE patients with LN [25, 26, 35].

A compelling body of evidence has shown that a combination of anti-C1q, anti-dsDNA, and/or nucleosome antibodies was strongly correlated with renal diseases and could be used for prognosis of patients with LN [6, 8, 11]. Furthermore, anti-C1q antibodies have been suggested to be more strongly correlated with renal flares compared to other serological markers [36], and patients free of anti-C1q antibodies are less likely to have active renal diseases [6, 10, 11, 37]. Given the fact that both serum anti-C1q and sIL-7R were strongly associated with SLE disease activity and LN, this may imply a correlation between the anti-C1q and sIL-7R, which may be a valuable diagnostic and prognostic marker for SLE and LN. Therefore, there is a need to further evaluate the correlation of anti-C1q and sIL-7R levels in sera of SLE patients in clinical settings. The objective of present report was first to determine associations of serum concentrations of anti-C1q antibodies and sIL-7R with LN and further evaluated a correlation between serum anti-C1q antibodies and sIL-7R of 134 SLE patients in a single center. Our results showed a strong association of serum anti-C1q or sIL-7R with renal disease activity in SLE patients, and these two serological markers also had a strong correlation in SLE patients with LN.

## 2. Materials and Methods

### 2.1. Ethics Statement

Human blood samples were collected with a protocol approved by the Ethic Committee for the Conduct of Human Research at Ningxia Medical University (NXMU-E2012-102p). Written consent was obtained from every individual according to the Ethic Committee for the Conduct of Human Research protocol. For the participants younger than 18 years, written inform consents were obtained from their guardians or parents on behalf of the children. All participants were provided a written informed consent for the publication of the data. The PI of this study maintains human research records, including signed and dated consent documents, for ten (10) years after the age of majority. The Ethic Committee the Conduct of Human Research at Ningxia Medical University approved the consent procedure for this study (NXMU-2012-102e).

### 2.2. Blood Samples

Blood samples of 134 consecutive SLE patient samples (118 females and 16 males) were collected from the outpatient rheumatology clinics of the General Hospital of Ningxia Medical University from January 2014 to June 2015. The mean ± SEM age for the SLE patients at the time of the sample drawn was 38.41 ± 1.14 years (range 12 to 68), with an average duration of diseases of 5.87 ± 0.84 (0.2 to 20 years). The American College of Rheumatology (ACR) criteria were used to diagnose a patient with SLE [38, 39], and the disease activity was defined according to SLE Disease Activity Index (SLEDAI) criteria [40, 41]. A patient with SLEDAI ≥10 was defined as active SLE. Renal involvement was defined based on clinical and laboratory manifestations. An active LN was defined as urine protein excretion ≥500 mg/day or cellular casts [38]. Sera of 84 gender and age-matched healthy individuals (6 males and 78 females) were also collected. These healthy control cohorts were recruited from those who had undergone comprehensive medical screening at the General Hospital of Ningxia Medical University and who had no history of chronic diseases and no family history of autoimmune diseases. The demographics of individuals involved in this study were outlined in Table 1. All sera were treated with heparin and frozen in 100 *μ*L aliquots at −80°C until being analyzed. There was no genetic relationship among these individuals. All the samples were collected under an informed consent.

### 2.3. Detection of Anti-C1q IgG Autoantibodies

The concentration of serum anti-C1q antibody was measured by an enzyme-linked immunosorbent assay (ELISA) using commercially available kits according to the manufacturer's instruction (INOVA Diagnostics Inc., San Diego, CA, USA) as previously described in our lab [6]. Briefly, sera were diluted 1/100 and then added into each well; the wells were washed with high ionic strength buffer after being incubated at room temperature for 1 h. Then, horseradish peroxidase coupled to anti-human IgG conjugate supplied with the kit was used as the secondary antibody. After 30 min incubation, the wells were extensively washed for three times, followed by the addition of 100 *μ*L trimethylbenzene solution and incubation for 30 min before 100 *μ*L of stopping solution was added into each well. The optical density was then measured at 450 nm. The absorbance (OD_450 nm_) was then converted into a concentration through standard curve with a cutoff value of 10 AU/mL (determined by the manufacturer). The cutoff values of anti-C1q in this study were <10 AU/mL, and ≥10 AU/mL was considered as positive as suggested by the manufacturer. Other laboratory data, including serum levels of complement C3, C4, and hemoglobin, antinuclear antibodies (ANA), anti-dsDNA antibodies, antiribonucleoprotein, perinuclear antineutrophil cytoplasmic antibody (pANCA), antibodies to Sjogren's syndrome A (SSA) and B (SSB), and anti-Smith (Sm), were also recorded, respectively (Table 1).

### 2.4. ELISA for sIL-7R

Serum sIL-7R concentration was determined using a biotin-avidin sandwich ELISA kit of human IL-7R according to the manufacturer's instruction (Elabscience Biotech, Wuhan, China). In this kit, the first anti-IL-7R antibody served as the capture antibody; the sIL-7R was detected with a biotinylated anti-IL-7R antibody generated in species other than that for producing the IL-7R capture antibody. Streptavidin-HRP was applied to determine the abundance of antigen-antibody binding as previously reported [33].

### 2.5. RNA Isolation and Real-Time Quantitative RT-PCR

The total RNA of peripheral blood mononuclear cells (PBMCs) was purified from whole blood using EasyPure Blood RNA kit per manufacturer's instruction (Transgen Biotech, Beijing, China). The quality of RNA was assayed by calculation of the RNA integrity number (RIN). High quality RNA (RIN value was greater than 9.0) was used for reverse transcription of first-strand cDNA synthesis by reverse transcription using M-MLV reverse transcriptase (TaKaRa, Dalian, China). The quantitative real-time RT-PCR (qRT-PCR) was performed in the Roche Lightcycler 2.0 using TaKaRa SYBR Green I kit (Takara, Dalian, China); the thermal cycling condition for PCR amplification was 95°C for 30 sec, 40 cycles of 95°C for 5 sec, 60°C for 20 sec, and 72°C for 20 sec, followed by 40°C for 20 min. The sequences of primer sets used for internal control *β*-actin and* sIL-7R* cDNA amplification were as follows: *β*-actin: forward: 5′AGCGAGCATCCCCAAAGTT3′ and reverse: 5′GGGCACGAAGGCTCATCATT3′; sIL-7R: forward: 5′GGATGTAGTCATCACTCCCAGAAAG3′ and reverse: 5′GGACCTGGAAGAGGAGAGAATA3′ [26]. An internal control was always included to normalize each reaction with respect to RNA integrity, sample loading, and inter-PCR variations. The relative expression ratio was calculated from the real-time PCR efficiencies and the crossing point deviation of* sIL-7R* gene against *β*-actin gene. The specificity of PCR was determined by sequencing of the PCR products.

### 2.6. Statistical Analysis

All laboratory data were entered into and extracted from PRISM (version 5) (GraphPad Software, La Jolla, CA, USA) and/or SPSS for Windows (version 17.0) (SPSS Inc., Chicago, IL, USA). Statistical evaluation of the data was performed by a* t-*test for comparison of differences between the two groups. The association between qualitative variables was evaluated by Spearman correlation. Data was presented as the mean ± standard error of mean (SEM). A *p* value of less than 0.05 was considered statistically significant. ^*∗*^ *p* < 0.05; ^*∗∗*^ *p* < 0.01; and ^*∗∗∗*^ *p* < 0.0001 (NS: no statistical difference).

## 3. Results

### 3.1. SLE Demographics Data

The unselected SLE population studied in this study included 118 s (86.76%) females and 16 (13.24%) males with a mean age of 38.41 ± 1.14 years (range 12 to 68), and the average duration of diseases was 5.87 ± 0.84 (0.2 to 20 years). The mean of SLEDAI score of SLE was 9.80 ± 0.65 (range 0 to 36). The data of demographics and other clinical parameters of SLE patients with LN and non-LN were presented in Table 1.

### 3.2. Serum Levels of sIL-7R and Anti-C1q Antibodies in SLE Patients

**Figure 1:**
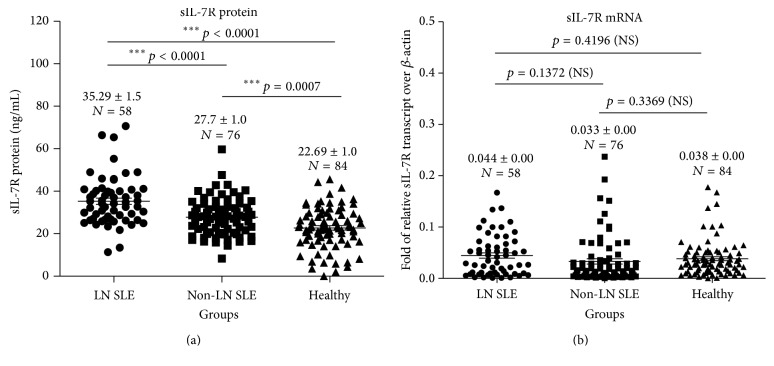
Increased soluble interleukin-7 receptor (sIL-7R) concentrations in sera of SLE patients with lupus nephritis (LN). (a) sIL-7R concentrations were measured by an ELISA in serum samples from SLE 58 patients with LN (LN-SLE) (*N* = 58), 76 SLE patients without LN (non-LN SLE), and 84 healthy individuals. (b) Quantitative PCR evaluation of the sIL-7R gene expression in peripheral blood mononuclear cells (PBMCs) collected from 136 SLE patients and 84 healthy individuals. *p* value by Student's *t*-test. Data presents as the mean ± SEM in each group. NS: no statistical difference (^*∗∗∗*^ *p* < 0.0001).

**Figure 2:**
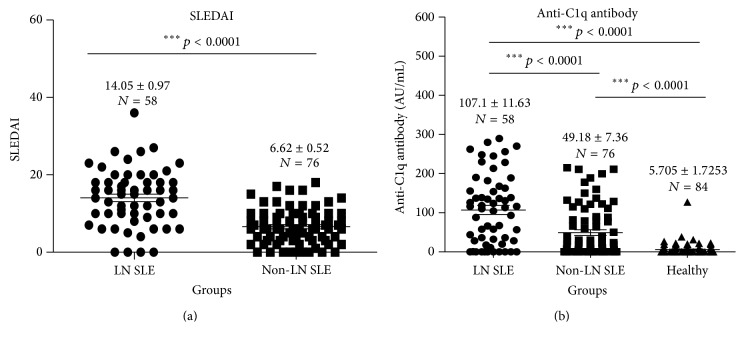
Higher SLE Disease Activity Index (SLEDAI) scores and serum anti-C1q antibody concentrations in SLE patients with lupus nephritis (LN) relative to non-LN SLE patients. (a) SLEDAI scores between LN SLE patients (*N* = 58) and non-LN SLE patients (*N* = 76). (b) Concentrations of anti-C1q antibody were measured by an ELISA in serum samples from SLE 58 patients with LN (LN-SLE) (*N* = 58), 76 SLE patients without LN (non-LN SLE), and 84 healthy individuals. *p* value by Student's *t*-test. Data presents as the mean ± SEM in each group.

Mounting evidence has revealed increased concentrations of sIL-7R and anti-C1q antibodies in sera of SLE patients, which were strongly associated with the disease activity of SLE and LN [6, 26, 35]. In line with these findings, an elevated sIL-7R was also determined in SLE patients with LN, as compared to non-LN SLE patients (35.29 ± 1.5 ng/mL versus 27.7 ± 1.0 ng/mL, *p* < 0.0001) and healthy cohorts (35.29 ± 1.5 ng/mL versus 22.69 ± 1.0 ng/mL, *p* < 0.0001) (Figure 1(a)). The serum concentration of sIL-7R in non-LN SLE patients was also higher in comparison with that in healthy controls (27.7 ± 1.0 ng/mL versus 22.69 ± 1.0 ng/mL, *p* < 0.0007) (Figure 1(a)). Interestingly, the abundance of sIL-7R transcript of PBMCs exhibited no statistical difference between these groups (Figure 1(b)), which was in agreement with the finding reported by Badot et al. [26]. Consistent with our previous findings [6], the SLEDAI scores and concentration of anti-C1q were greater in LN SLE patients than those in non-LN SLE patients and healthy individuals (Figure 2). The average SLEDAI scores in SLE patients with LN versus SLE patients without LN were (14.05 ± 0.97 versus 6.62 ± 0.52, *p* < 0.0001) (Figure 2(a) and Table 1). The titer of anti-C1q antibody in SLE patients with LN versus SLE patients without LN was 107.1 ± 11.63 AU/mL versus 49.18 ± 7.36 AU/mL, *p* < 0.0001; in SLE patients without LN versus healthy individuals was 49.18 ± 7.36 AU/mL versus 5.705 ± 1.73 AU/mL, *p* < 0.0001; and in SLE patients with LN versus healthy cohorts was 107.1 ± 11.63 AU/mL versus 5.705 ± 1.73 AU/mL, *p* < 0.0001 (Figure 2(b) and Table 1).

### 3.3. Serum Levels of Complements C3 and C4, Anti-dsDNA, and Antinuclear Antibody in SLE Patients

**Figure 3:**
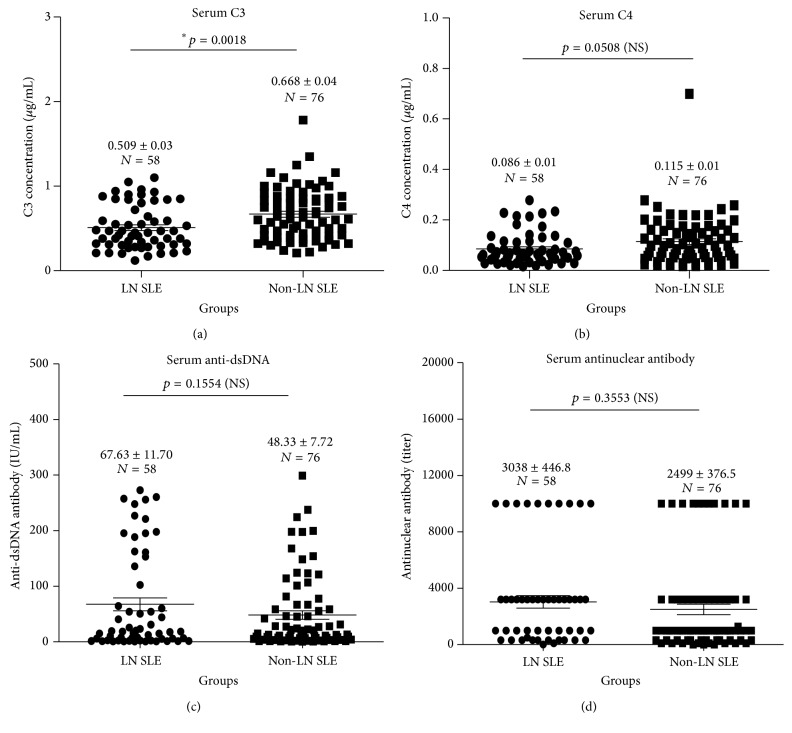
Levels of serum C3, C4, anti-dsDNA antibody, and antinuclear antibody (ANA) between SLE patients with LN and SLE patients without active LN. (a) Serum C3 concentration between LN SLE patients and non-LN SLE patients. (b) Serum C4 concentration between LN SLE patients and non-LN SLE patients. (c) Concentration of serum anti-dsDNA between LN SLE patients and non-LN SLE patients. (d) Concentration of serum antinuclear antibody between LN SLE patients and non-LN SLE patients. *p* value by Student's *t*-test. Data presents as the mean ± SEM in each group. NS: no statistical difference.

Serum concentrations of complements C3 and C4 were lower in patients with LN as compared with those without LN disease (Figures 3(a) and 3(b), Table 1). The C3 concentrations between SLE patients with LN and without LN were 0.51 ± 0.03 *μ*g/mL and 0.69 ± 0.04 *μ*g/mL, respectively (*p* = 0.0018) (Figure 3(a)); the C4 concentrations between SLE patients with LN and without LN were 0.086 ± 0.01 *μ*g/mL and 0.115 ± 0.01 *μ*g/mL, respectively (*p* = 0.0508) (Figure 3(b)). Antibodies to ds-DNA and antinuclear antibody (ANA) were the most prevalent autoantibodies observed in these SLE cohorts as determined by ELISA, which were detected in 100% (134/134) and 97.02% (130/134) of SLE patients, respectively (Table 1). In line with the concentrations of anti-C1q antibodies detected in SLE, the titers of anti-dsDNA and ANA were moderately higher in SLE patients with LN as compared with those without a renal involvement, but there was no statistical difference determined in this study, respectively (Figures 3(c) and 3(d), Table 1). The titers of anti-dsDNA antibodies were 67.63 ± 11.70 IU/mL in the SLE with LN and 48.33 ± 7.72 IU/mL in the SLE without LN (*p* = 0.1554) (Figure 3(c)); the titers of ANA were 3038 ± 446.8 in the SLE with LN and 2499 ± 376.5 in the SLE without LN (*p* = 0.3553) (Figure 3(c)). Other autoantibodies, including antibodies to cardiolipin (ACL), cytoplasmic antineutrophil cytoplasmic antibody (cANCA), perinuclear neutrophil cytoplasmics (pANCA), ribosomal P-proteins (Rib-P), ribonucleoprotein, and Sjogren's syndrome A and B, were also detected in SLE patients, which were listed in Table 1. Of note, significant differences between SLE patients with LN and non-LN were only observed in serum levels of sIL-7R, anti-C1q, and complement C3 in this study.

### 3.4. Correlations of SLEDAI Scores of sIL-7R and Other Serological Biomarkers

**Figure 4:**
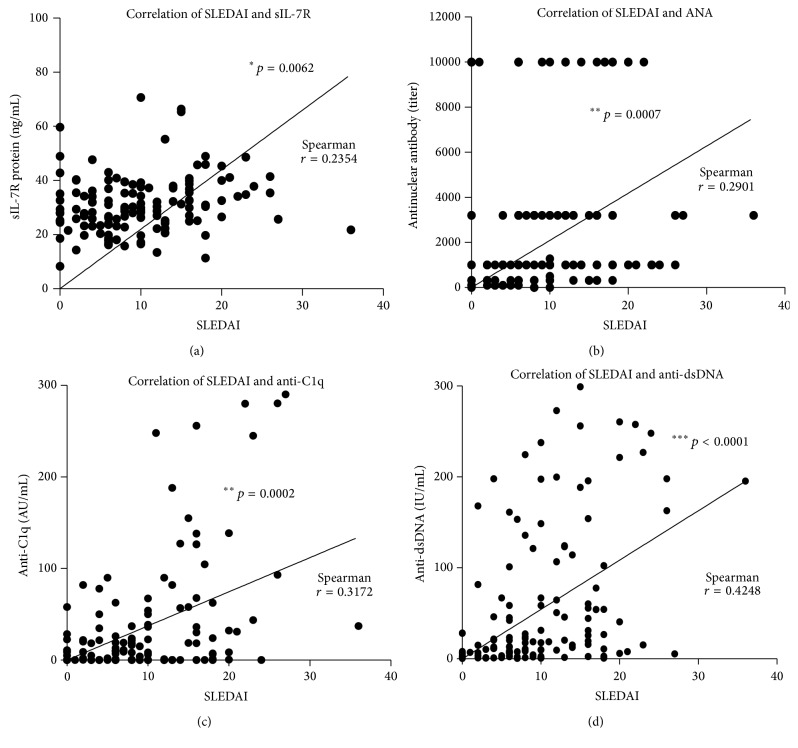
Correlation between SLEDAI scores and serum concentrations of sIL-7R, anti-nuclear antibody, anti-C1q antibody, and anti-dsDNA antibody in SLE patients. (a) Correlation between SLEDAI scores and the serum concentration of sIL-7R in SLE patients (*N* = 136). (b) Correlation between SLEDAI scores and the concentration of serum antinuclear antibody in SLE patients (*N* = 136). (c) Correlation between SLEDAI scores and the concentration of serum anti-C1q antibody in SLE patients (*N* = 136). (d) Correlation between SLEDAI scores and the serum concentration of anti-dsDNA antibody in SLE patients (*N* = 136). Spearman *r* and *p* values are displayed in each graph. *p* value by two-tailed Pearson correlation test.

In order to reveal the clinical significances of circulating biomarkers in SLE, the correlations between SLEDAI scores and several serological biomarkers, including sIL-7R, were evaluated (Figure 4). The correlation coefficients between SLEDAI scores and sIL-7R, ANA, anti-C1q antibodies, and anti-dsDNA antibodies were *r* = 0.2354 (*p* = 0.0062) (Figure 4(a)), *r* = 0.2901 (*p* = 0.0007) (Figure 4(b)), *r* = 0.3172 (*p* = 0.0002) (Figure 4(c)), and *r* = 0.4248 (*p* < 0.0001) (Figure 4(d)), respectively. Of interest, the concentrations or titers of these serum markers had statistically significant correlations with SLEDAI scores.

### 3.5. A Positive Correlation between Serum sIL-7R and Anti-C1q Antibodies in SLE Patients

**Figure 5:**
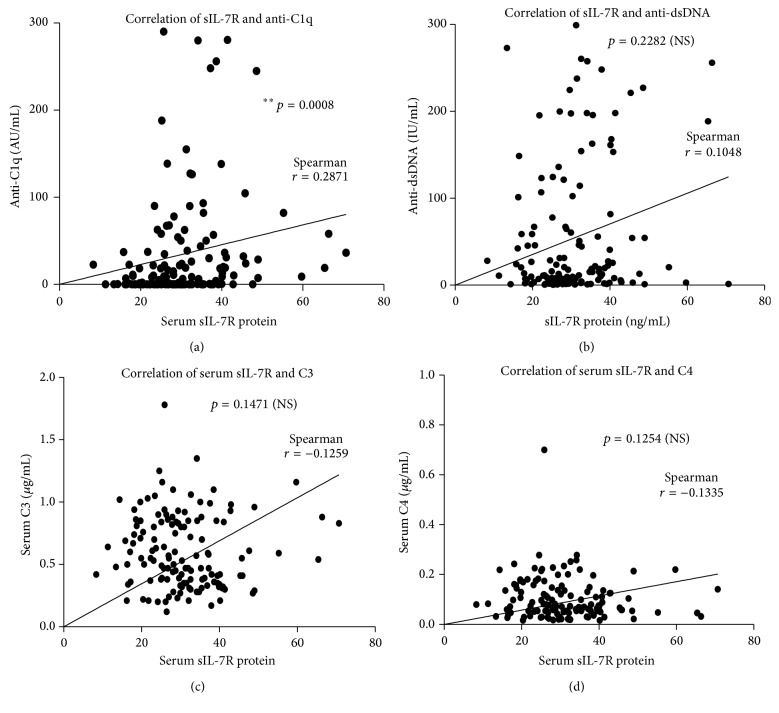
Correlation between serum concentrations of sIL-7R and serum concentrations of anti-C1q antibody, anti-dsDNA antibody, C3, and C4 in SLE patients. (a) Correlation between serum sIL-7R concentration and the level of anti-C1q antibody in SLE patients (*N* = 136). (b) Correlation between serum sIL-7R concentration and the level of anti-dsDNA antibody in SLE patients (*N* = 136). (c) Correlation between serum sIL-7R concentration and the C3 concentration in SLE patients (*N* = 136). (d) Correlation between serum sIL-7R concentration and the C4 concentration in SLE patients (*N* = 136). Spearman *r* and *p* values are displayed in each of the graphs. *p* value by two-tailed Pearson correlation test. NS: no statistical difference.

We next sought to analyze whether the sIL-7R correlated with other serological biomarkers. The correlation coefficients between sIL-7R and anti-C1q antibodies, anti-dsDNA antibody, and C3 and C4 concentrations were *r* = 0.2871 (*p* = 0.0008) (Figure 5(a)), *r* = 0.1048 (*p* = 0.2282) (Figure 5(b)), *r* = −0.1259 (*p* = 0.1471) (Figure 5(c)), and *r* = −0.1335 (*p* = 0.1254) (Figure 5(d)), respectively. Of interest, only the anti-C1q antibodies showed a statistically significant association with sIL-7R (Figure 5(a)). There was no significant association detected between the sIL-7R and serological biomarkers other than the anti-C1q antibodies. ANA also had no correlation with sIL-7R (data not shown). These data imply that a combination of anti-C1q antibodies and sIL-7R may enhance the specificity in the identification of patients with active SLE and LN.

## 4. Discussion

The sIL-7R is a novel circulating biomarker that has diagnostic and prognostic values for disease activity and renal flares in SLE patients. In this report, we evaluated the serum concentrations of sIL-7R and anti-C1q autoantibodies and analyzed correlations of sIL-7R with SLE disease activity (SLEDAI scores) and other serological biomarkers in 134 SLE patients. The results showed that both sIL-7R and anti-C1q were strikingly elevated in patients with active SLE and LN relative to patients with inactive SLE and non-LN, and healthy control individuals. In addition, the serum levels of sIL-7R and anti-C1q antibodies were positively correlated with SLEDAI scores in SLE patients. Interestingly, the sIL-7R displayed a strong association with serum anti-C1q antibodies in SLE patients, implying that both of them may be novel biomarkers in SLE, and a combination of sIL-7R and anti-C1q antibodies, or other serological biomarkers, may increase the diagnostic specificity for identification of patients with active SLE or LN. Such observation is consistent with findings from other groups [6, 10, 12, 14, 23, 26, 35].

Since the involvement of renal flare in SLE diseases represents a major complication in the treatment, an early identification of LN would guide an early intervention for rheumatologists in a clinical setting. A compelling body of studies has indicated that the anti-dsDNA and anti-Sm antibodies are useful serological marker for identifying active SLE and LN activity [42]. However, different assays of anti-dsDNA antibodies and complements C3 and C4 have significant impacts on diagnosing SLE disease activity in terms of the sensitivity and specificity [16]. Such variations in serological indices of systemic disease activity do not accurately reflect the activity of SLE. These biomarkers are not necessarily associated with active renal disease, although they may be a high predictive negative value in SLE [43]. In addition, although the presence of renal-specific haematuria and the quantification of proteinuria are apparently associated with the presence of glomerular lesions, they may be from a consequence of glomerular damage rather than inflammation. A histological evaluation of repeat-biopsy specimens is thus usually required for assessment of renal disease activity in SLE.

Recently, an elevated level of antibodies to C1q was frequently observed in the sera of patients with active SLE and LN, which was strongly associated with the hypocomplementemia and development of LN, and SLE patients free of these antibodies were very unlikely to have active renal flares [2, 3, 6, 10, 44–46]. Mechanistically, an elevated anti-C1q may induce the formation of C1q-anti-C1q complexes and promote the production of inflammatory mediators, which in turn inhibits the activation of complement and the clearance of immune complexes, sequentially results in further release of autoantigens, production of autoantibodies, and formation of complexes, eventually activates diseases, and leads to tissue damage [47]. With respect to hypocomplementemia, the anti-C1q can activate the classical pathway and lectin pathway but not the alternative pathway of complement, depending on the anti-C1q immunoglobulin-class repertoire present in the sera of SLE patients, suggesting an important role of anti-C1q in SLE hypocomplementemia [48]. In the present study, an elevated level of anti-C1q antibodies was also detected in patients with active SLE and LN, in comparison with healthy cohorts, and those with inactive SLE and nonrenal involvement. This finding supports the view of the fact that anti-Clq antibodies alone or in combination with other serological markers can be used as an important diagnostic parameter for identifying SLE patients with active disease and LN [2, 3, 6, 8, 10, 11, 15].

Apart from anti-C1q autoantibodies, certain cytokines may also serve as serological markers to monitor disease activity and predict disease severity. Among these serum cytokines, sIL-7R has recently spurred an increased interest as a serological marker, owing to its strong association with autoimmune diseases and the activity of renal flares in SLE patients [24, 26, 31, 35]. In the current context, an increased circulating sIL-7R concentration can potentiate IL-7 bioactivity and promote autoimmunity* in vivo*, through a mechanism by which the sIL-7R is able to compete with cell-associated IL-7 receptor and diminish excessive IL-7 consumption, sequentially enhances proliferative responses of T-cells to weak self-antigens, and leads to autoimmune diseases, such as type I diabetes, RA, MS, and SLE [24, 25, 30]. This notion was further supported by polymorphic analysis in human MS and SLE, in which polymorphisms of IL-7R were associated with the susceptibility to autoimmune diseases, such as SLE [24, 34].

With respect to the concentration of circulating sIL-7R, it was observed to be elevated in synovial tissue and sera of RA patients [31, 49, 50] and patients with MS [24] and SLE [26, 35]. Importantly, the level of serum sIL-7R was found to be strongly correlated with the disease activity and renal flares in SLE patients [26, 35], which was consistent with a finding in RA patients, in whom an increased serum sIL-7R concentration was associated with poor response to (methotrexate and TNF-blocking) therapy [31]. In the present study, a significantly higher level of sIL-7R was also detected in sera of SLE patients with LN, in comparison with non-LN patients, which was also positively correlated with the disease activity as determined by SLEDAI scores. These studies and ours suggest that the serum sIL-7R may be a unique surrogate marker for accessing renal flares in SLE patients. Furthermore, a combination of sIL-7R and other biomarkers such as anti-C1q, anti-dsDNA, and/or complements C3 and C4 may increase the specificity for identification of active LN in SLE patients with complex disease manifestations [35]. Particularly, the titer of anti-C1q was observed to positively correlate with serum concentration of sIL-7R in this study, implying that a combination of sIL-7R and anti-C1q may enhance the diagnostic and prognostic specificity for LN using serological biomarkers in clinical settings, which warrants further investigation.

Interestingly, the abundance of IL-7R transcript was not statistically altered in PBMCs from patients with LN compared with those without LN and control individuals, which was in disagreement with its protein concentration detected in sera but was in line with the finding reported by Badot et al. [26]. Together with expression of IL-7R in kidney perivascular cells, this observation may indicate that an elevated concentration of sIL-7R in sera of patients with LN reflects activation of renal cells [26].

## 5. Conclusions

Collectively, this study in 134 SLE patients further confirms a previous finding of a correlation of serum sIL-7R concentration with SLE disease activity and LN. Intriguingly, serum levels of sIL-7R were positively correlated with the abundances of anti-C1q antibodies in SLE patients. This study thus supports a view of the fact that sIL-7R is a unique serological marker for SLE disease activity and LN, and a combination of sIL-7R and other markers, such as anti-C1q, may increase the specificity for assessment of disease activity in SLE patients in clinical settings. Limitations of this study include the fact that only a small size of SLE samples was studied, and follow-up data were also lacking; the LN activity was mainly determined by laboratory parameters and clinical manifestations rather than by pathogenic analysis in renal biopsies.

## Figures and Tables

**Table 1:** Demographics of patients with systemic lupus erythematosus (SLE) (*N* = 134).

Demographics	LN SLE	Non-LN SLE	*p*
Patient number (%)	58/134 (43.28)	76/134 (56.72)	NA
Age (mean ± SEM) (range, years)	37.34 ± 1.55 (18–65)	39.27 ± 1.59 (12–68)	0.2359
Gender (male/female) (% female)	6/52 (89.66)	10/66 (86.84)	NA
Disease duration (mean ± SD) (range, years)	6.23 ± 0.56 (0.5–20)	5.30 ± 0.79 (0.2–18)	0.3768
SLEDAI score (range)	14.05 ± 0.97 (0–36)	6.62 ± 0.52 (0–18)	<0.0001^*∗∗∗*^
ACL Ab (+) number (%)	42/58 (72.41)	48/76 (63.18)	NA
Anti-C1q (+) number (%)	49/58 (84.48)	31/76 (40.79)	NA
Anti-C1q (AU/mL)	78.63 ± 16.87	26.88 ± 9.236	<0.0001^*∗∗∗*^
Anti-dsDNA (+) number (%)	58/58 (100)	76/76 (100)	NA
Anti-dsDNA (IU/mL)	67.63 ± 11.70	48.33 ± 7.721	0.1554
ANA (+) number (%)	57/58 (98.28)	73/76 (96.05)	NA
ANA titer	3038 ± 466.2	2499 ± 376.5	0.3553
Anti-Rib-P (+) number (%)	12/58 (20.69)	12/76 (15.79)	NA
Anti-Smith (Sm) (+) number (%)	18/58 (31.03)	15/76 (19.74)	NA
Anti-SSA Ab (+) number (%)	28/58 (48.28)	27/76 (35.53)	NA
Anti-SSB Ab (+) number (%)	15/58 (25.86)	10/76 (13.16)	NA
pANCA (+) number (%)	16/58 (27.59)	17/76 (30.36)	NA
cANCA (+) number (%)	1/58 (1.72)	0/76 (0.00)	NA
C3 (*µ*g/mL)	0.5091 ± 0.0340	0.6680 ± 0.03485	0.0018^*∗*^
C4 (*µ*g/mL)	0.08557 ± 0.0082	0.1145 ± 0.0112	0.0508

Ab: antibody; ACL: anticardiolipin; ANA: antinuclear antibody; cANCA: cytoplasmic antineutrophil cytoplasmic antibody; LN: lupus nephritis; pANCA: perinuclear antineutrophil cytoplasmic antibody; Rib-P: ribosomal P-proteins; RNP: ribonucleoprotein; SSA: Sjogren's syndrome A; SSB: anti-Sjogren's syndrome B.

^*∗∗∗*^ *p* < 0.0001; and ^*∗*^ *p* < 0.01.
